# CRISPR-Cas9-driven antigen conversion of clinically relevant blood group systems

**DOI:** 10.1093/hmg/ddaf040

**Published:** 2025-04-02

**Authors:** Yelena Boccacci, Nellie Dumont, Yannick Doyon, Josée Laganière

**Affiliations:** Centre Hospitalier Universitaire de Québec Research Center - Université Laval, 2705 boulevard Laurier, Québec, QC G1V 4G2, Canada; Faculty of Medicine, Laval University, 1050 avenue de la Médecine, Québec, QC G1V 0A6, Canada; Université Laval Cancer Research Centre, 1050 avenue de la Médecine, Québec, QC G1V 0A6, Canada; Medical Affairs and Innovation, Héma-Québec, 1070 avenue des Sciences-de-la-Vie, Québec, QC G1V 5C3, Canada; Medical Affairs and Innovation, Héma-Québec, 1070 avenue des Sciences-de-la-Vie, Québec, QC G1V 5C3, Canada; Centre Hospitalier Universitaire de Québec Research Center - Université Laval, 2705 boulevard Laurier, Québec, QC G1V 4G2, Canada; Faculty of Medicine, Laval University, 1050 avenue de la Médecine, Québec, QC G1V 0A6, Canada; Université Laval Cancer Research Centre, 1050 avenue de la Médecine, Québec, QC G1V 0A6, Canada; Faculty of Medicine, Laval University, 1050 avenue de la Médecine, Québec, QC G1V 0A6, Canada; Medical Affairs and Innovation, Héma-Québec, 1070 avenue des Sciences-de-la-Vie, Québec, QC G1V 5C3, Canada

**Keywords:** hematopoietic stem and progenitor cells, erythroid progenitors, blood groups, CRISPR-Cas9, gene editing

## Abstract

The common practice of blood transfusion entirely relies on blood donations from the population. Ensuring blood group compatibility between a donor and a recipient is paramount to prevent critical adverse reactions. Finding compatible blood can be challenging given the high diversity of blood group antigens, especially for chronically transfused patients at higher risk of alloimmunization owing to repeated exposures to foreign RBCs. In addition, due to the immunogenicity of the ABO blood group and the highly polymorphic nature of the Rhesus (Rh) system, they both remain of prime importance in transfusion medicine. Cultured red blood cells (cRBCs) may eventually provide an alternative for blood donations—at least in some circumstances. Combining cRBCs with blood group gene editing could broaden transfusion accessibility by making antigen expression compatible with rare phenotypes, thus meeting the needs of more patients. Starting from mobilized, erythroid-primed hematopoietic stem and progenitor cells (HSPCs), we used virus- and selection-free, CRISPR-Cas9-mediated knockouts to produce erythroid cells devoid of AB and Rh antigen. The approach yielded almost complete conversion to O- and RhNull phenotypes, as determined by standard hemagglutination and flow cytometry analyses. Combined with robust cRBC protocols, these clinically relevant phenotypic changes could eventually expand the accessibility of blood transfusion for specific and unmet clinical needs.

## Introduction

Blood transfusions are essential to manage the symptoms of many patients, including those with cancer undergoing chemotherapy, and those with acquired or hereditary blood disorders affecting red blood cells (RBCs) or the bone marrow (e.g. sickle cell disease [SCD]) [1]. However, blood availability [2, 3], transfusion reactions, and blood group incompatibilities often limit their safety and applicability [4, 5].

When the immune system encounters new, foreign RBCs expressing non-self antigens, alloantibodies can be produced and trigger an hemolytic reaction upon reexposure [4, 5]. This concern is especially relevant for chronically transfused patients, such as those with SCD, who are frequently exposed to foreign RBCs and present a pro-inflammatory state that promotes alloimmunization [6–8]. For these patients, preventing alloimmunization through rigorous matching of the major blood group systems is critical for long-term disease management.

Forty-seven blood group systems have been identified so far [9, 10] (see also ISBT Blood Group Allele Tables https://www.isbtweb.org/isbt-working-parties/rcibgt/blood-group-allele-tables.html), but the Rh and ABO systems stand out as the most clinically relevant owing to the immunogenicity of their associated antigens [11, 12].

The ABO blood group system is composed of the A, B and H carbohydrate antigens. The H antigen is formed when the Fut1 fucosyltransferase transfers a fucose on type 2 glycoproteins on all RBCs. It defines the O blood group while being the precursor for the A and B antigens/blood groups. Mutations in the H (*FUT1)* gene can deplete the H antigen levels and cause the rare Bombay phenotype (1 in 4 000 000 persons). The A and B alleles each encode a glycosyltransferase that catalyses the final step of the A or B antigen conversion from the H antigen. Two single-base substitutions are responsible for the specificity between the A- and B-transferases, while a single nucleotide deletion causes a non-functional enzyme and hence the majority of the O-type alleles [13–19].

The Rh blood group system is much more complex as it comprises over 50 (i.e. 56 to date) antigens encoded by the homologous *RHD* and *RHCE* genes. This complexity stems from numerous single nucleotide polymorphisms (SNPs), deletions, as well as recombination products between *RHD* and *RHCE* [14, 20]. The polymorphic nature of the *RH* genes makes challenging the search for units compatible for Rh antigens, particularly across cultural communities [21–23].

The Rh_null_ phenotype is a rare blood group characterized by the complete absence of Rh D, C and E antigens at the cell surface. It is therefore compatible with all Rh blood types when used as donor cells (in contrast to the Rh- (or RhD-) blood group which is only compatible when used as donor cells for all RhD variants). The genotypes that confer Rh_null_ belong to the amorph type when silencing mutations in *RHD* and *RHCE* thwart their expression [24, 25], and to the regulatory type when *RHAG* mutations thwart the formation and membrane assembly of the RHD/RHCE/RHAG complex [26]. Other membrane proteins (e.g. LW and CD47) bridge the Rh complex with the cytoskeleton [27]. Rh_null_ RBCs are thought to display decreased detectability of associated proteins like CD47, attributed to potentially slightly altered membrane integrity [28–30].

Since Rh_null_ blood is fully compatible with all other Rh phenotypes [31], it is of potential high interest in the transfusion medicine world. However, this ‘golden blood’ is exceedingly rare in the general population (1 in 6 000 000 persons) thus the probability of obtaining Rh_null_ blood through regular donations is scarce [32].

The production of *in vitro* cultured RBCs of enhanced compatibility has long been a goal in transfusion medicine. To produce these cells, the hematopoietic stem and progenitor cells (HSPCs) of a donor could be isolated and cultured into RBCs in the laboratory. Various types of cell lines such as immortalized erythroblasts, iPSCs, etc. could also be derived from rare blood donors, but technical limitations are still a hurdle as far as their ability to efficiently produce mature RBCs *in vitro* [33].

Alternatively, the genotypes conferring the desired phenotype could be implemented through gene transfer or editing. A few reports describe blood group modification in immortalized cell lines using lentiviral vector and selection-based gene knockdown (shRNA), or knockout with the CRISPR-Cas9 [34, 35] and TALEN systems [36]. HSPC have been extensively used to produce RBCs *in vitro* [37–40]. Although not a renewable cell source, given their advantage of being more efficient at producing mature RBCs compared to immortalized erythroid cell lines, they remain an interesting cell source that might reach the clinic first for some applications [40]. Although HSPCs can be efficiently edited using the CRISPR-Cas9 system [41–44], RBC blood groups have only been modified taking advantage of viral delivery methods followed by selection strategies to reach gene expression knockdown with shRNA [28] or overexpression using cDNA [45]. It is thus worth exploring whether HSPCs could be exploited for their current RBC large-scale production methods destined to clinical use, and combined with gene editing, with a preference for viral-free gene modification workflow. Gene-modified lab-grown cRBCs could then be envisioned for patients with specific needs, for instance chronically transfused, allo-immunized patients, and those with rare blood. The resulting gene-edited RBCs could also serve as control reagents for serological diagnostic laboratories, which often rely on blood donations for RBCs with rare phenotypes (e.g. Rh_null_) that are not commercially available [46, 47].

In this work, we used CRISPR-Cas9-mediated gene ablation to produce [1] regulatory-type Rh_null_ erythroid cells starting from Rh + HSPCs, and [2] group O erythroid cells starting from group A HSPCs. The resulting cells yielded near-complete absence of respective antigen at their surface, which was ascertained by hemagglutination tests and flow cytometry analyses. Our study therefore offers an approach that might eventually complement blood transfusion to address incompatibility issues.

## Results

### Knockout of *RHAG* in HSPCs to produce Rh_null_ erythroid cells

**Figure 1:**
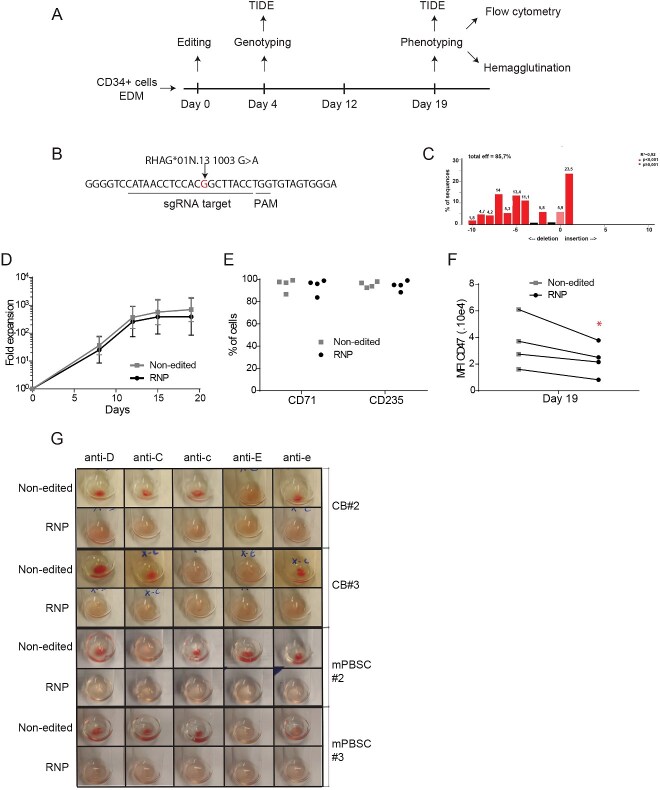
Production of Rh_null_ erythroid cells from Rh + HSPCs using a selection-free, CRISPR-Cas9-based approach. CB HSPCs from two donors (CB #2: Rh blood groups DCcee; CB#3 Rh blood group DCCee), and mPBSCs from two donors (mPBSC#2, Rh blood groups DccEE; mPBSC#3, Rh blood group DCcee) were used in these experiments. HSPCs were cultured in EDM and were submitted to nucleofection with or without the RNP targeting a *RHAG* sequence that overlaps a Rh_null_-associated SNP. (A) Protocol timeline; (B) sequence targeted by the sgRNA, with the SNP RHAG*01 N.131003 G > a highlighted; (C) A representative example of the NHEJ efficiency obtained at day 4, as assessed by TIDE; (D) Mean cumulative fold expansion up until day 19; (E) Percentage of CD71+ and CD235+ cells obtained by flow cytometry at day 19 for each donor; (F) Mean fluorescent unit (MFI) of CD47 levels at day 19, for each donor. *P* = 0.05 (*); (G) Photos of hemagglutination tests performed for RNP-free and RNP-treated HSPCs at day 19 of erythroid culture.

We first aimed to design a CRISPR-Cas-9 ribonucleoprotein complex targeting the *RHAG* gene coding region. It is expected that the double-strand break induced by the RNP will create indels resulting from the non-homologous end joining repair mechanism (NHEJ). This absence of RhAG is then expected to translate into an Rh_null_ phenotype. The strategy mimics a natural phenomenon and has been exploited by others in different settings, either using lentiviral delivery of RHAG-shRNAs into HSPCs or CRISPR-Cas9 modification of iPSCs or BelA immortalized erythroblasts [28, 34, 35]. First, five sgRNAs were tested by transfecting as an RNP complex in CB HSPCs (CB#2): this HSPC source was used for sgRNA screenings since readily accessible. Targeting efficiencies were analyzed by sequencing and quantification of NHEJ event using the TIDE software 4 days after transfection. Three (i.e. ‘RHAG_1’, ‘RHAG_2’, and ‘RHAG_3’) were designed to target *RHAG* sequences that overlap an Rh_null_-causing SNP (i.e. RHAG*01 N.131003 G > A) [14]. In addition, one was tested in a previous publication (i.e. ‘RHAG_4’) [35], and one targeted a sequence nearby that of RHAG_4 (i.e. ‘RHAG_5’). RHAG_2 was selected for subsequent experiments because it exhibited the highest efficiency and displayed the least amounts of in-frame modifications (Fig. 1B, Fig. S1).

To test the capacity to engineer HSPC-derived Rh_null_ cRBCs, erythroid-primed HSPCs from two CB and two mPBSC donors underwent the procedure outlined in (Fig. 1A). We used the two cell sources in parallel since they behave slightly differently in erythroid differentiation conditions and relative antigen expression levels may differ in derived cRBCs. Cells from a variety of extended Rh blood groups were used: CB donors #2 and #3 beared the DCcee and DCCee Rh blood group phenotypes, while the mPBSC donors #2 and #3 corresponded to DccEE and DCcee phenotypes, respectively.

Briefly, cells were transfected with or without RNP and cultured in erythroid differentiation medium (EDM) for 19 days. At day 4, up to 86% of alleles contained indels, of which at least 75% are expected to induce frameshifted transcripts (Fig. S2), and a representative example of the TIDE result is shown in (Fig. 1C). This high editing efficiency was maintained throughout the culture in the samples tested, as reflected by the day 15 and day 19 TIDE results (Fig. S2). Both RNP-free and RNP-treated cells had similar fold expansions at day 19 (Fig. 1D). RNP-treated cells differentiated normally, with > 90% cells expressing the CD71 and CD235a erythroid markers at day 19 (Fig. 1E). Interestingly, RNP-treated cells had 37.5% lower levels of CD47 at their cell surface (as measured by mean fluorescent intensity [MFI]) than RNP-free cells at day 19 (*P* = 0.050;Fig. 1F), thus recapitulating the phenomenon seen in RBCs of individuals bearing Rh_null_ phenotypes [28–30]. Finally, in order to functionally characterize the respective phenotypes, edited and non edited cRBCs derived from CB (*n* = 2) and adult mobilized (*n* = 2) HSPCs were submitted to hemagglutination tests that are routinely performed in blood banks. In all RNP-transfected conditions, cRBCs did not agglutinate in the presence of anti-D, and-C, anti-c, anti-E or anti-e, despite their original respective Rh-positive phenotypes (also shown as control in the ‘non-edited’ samples) (Fig. 1G). These results are thus compatible with Rh_null_ cRBCs and support a high-efficiency editing of HSPCs, as well as an efficient Rh complex inactivation. These results demonstrate the feasibility of generating Rh_null_ erythroid cells through CRISPR-Cas9-mediated targeting of homozygous wild type *RHAG* in HSPCs.

### Knockout of the *ABO* gene in erythroid-primed HSPCs to produce group O erythroid cells

**Figure 2:**
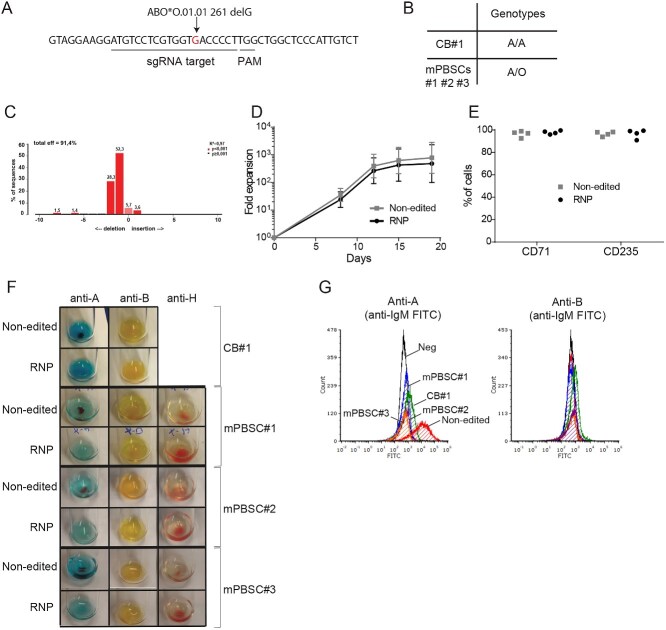
Production of group O erythroid cells from group A HSPCs using a selection-free, CRISPR-Cas9-based approach. Group A CB-derived HSPCs (CB #1) and mPB-derived HSPCs (mPBSC #1–2-3) were used in these experiments. HSPCs were cultured in EDM and transfected with or without the RNP targeting an *ABO* sequence that overlaps an O-associated SNP. (A) Sequence targeted by the sgRNA, with the ABO*O.01.01261delG SNP highlighted; (B) Genotypes of CB and mPBSC donors; (C) Representative example of the NHEJ efficiencies obtained, as assessed by TIDE at day 4; (D) Mean cumulative cell expansion up until day 19; (E) Percentage of CD71+ and CD235+ cells obtained by flow cytometry at day 19, for each donor; (F) Photos of hemagglutination tests performed for RNP-free and RNP-treated HSPCs at day 19 of erythroid culture; (G) Flow cytometry analyses of A and B antigen expression at day 19 of culture.

To broaden the applicability of this approach for other blood group systems, we next aimed at engineering group O erythroid cells from HSPCs. Our strategy was to design sgRNAs targeting the *ABO* locus overlapping a coding SNP responsible for causing an O phenotype the most frequently (ABO*O.01.01261 del G) [14]. Four *ABO*-targeting sgRNAs were tested in CB-derived HSPCs; results of the two most active ones are shown in (Fig. S3). ABO_2 sgRNA was selected for the following experiments based on its predicted binding specificity in the human genome and its high efficiency (target site shown in Fig. 2A).

HSPCs from group A donors underwent the procedure described in (Fig. 1A). Erythroid-primed HSPCs from one CB donor (A/A genotype) and three mPBSC donors (all of A/O genotype) were transfected with or without RNP and cultured 19 days in EDM (Fig. 2B)**.** Up to 91% of alleles contained indels at day 4, of which at least 87% are predicted to induce frameshift and knockout (Fig. 2C, Fig. S4). This high editing efficiency was also maintained throughout the culture (until days 15 and 19; Fig. S4). Both the RNP-free and the RNP-treated cells had similar fold expansions at day 19 (Fig. 2D). Additionally, the RNP-treated cells differentiated normally, with > 90% of cRBCs expressing the erythroid markers CD71 and CD235a (Fig. 2E).

Edited and non-edited cRBCs were then evaluated functionally through hemagglutination test using anti-A, anti-B and anti-H antibodies, the latter expected to react against the H antigen expressed by type O RBCs. At day 19 of erythroid culture, no hemagglutination was present in edited cells when anti-A antibodies were added, in contrast to the related non edited cells (Fig. 2F). Conversely, anti-H induced agglutination of the non-edited, donor-derived cRBCs of A/O genotype, as expected since present on group O cells and not on A or B group RBCs. Interestingly, in the RNP-treated cells, the strength of the anti-H reaction was more pronounced in all donors, reflecting the higher levels of H antigen exposure at the surface of the newly formed group O cRBCs (Fig. 2F). Flow cytometry was then used to attempt surface antigen quantification. In CB-derived cRBCs (genotype: A/A), 77% of the non-edited cRBCs displayed the A antigen, which decreased to 16% after editing (Fig. 2G). While the A antigen was detected on 80% of cRBCs derived from non edited adult-derived HSPCs (genotype: A/O), only 2% of cRBCs expressed it when derived from edited HSPCs (Fig. 2G). As expected from the donor’s blood groups, the B antigen was not detected in any of the edited or non-edited cRBCs (Fig. 2G).

We then explored whether an HDR-based strategy could further improve the efficiency of gene ablation. Specifically, a single-stranded DNA containing the most common O-associated SNP (ABO*O.01.01261 del G) [14] served as a repair template for CB-derived cells (genotype: A/A) and was co-transfected with the RNP. This HDR-based strategy achieved a high efficiency (HDR [measured by RFLP]: 30%; NHEJ: 62%; Fig. S5A). While no anti-A hemagglutination was observed in edited cRBCs (Fig. S5B), flow cytometry showed a residual expression of the A antigen on 18% of the cells (Fig. S5C), which is equivalent to the strategy without added donor DNA (which reached 16% A-antigen expressing cRBCs). This HDR-based strategy thus seemingly offered no benefit over the NHEJ-based strategy.

Together, these results demonstrate the feasibility of efficiently generating group O erythroid cells by CRISPR-Cas9-mediated knockout of *ABO* in HSPCs.

## Discussion

Genetically engineering cRBCs of defined blood groups would be a welcome addition in transfusion medicine, whether it be for therapeutic use or the development of serological tools. Here, we demonstrate the potential of CRISPR-Cas9-mediated gene knockout to produce erythroid cells with phenotypes of interest, using the rare Rh_null_ phenotype as a proof of concept. We further demonstrate the versatility of the approach with other blood group systems by producing *ABO* knockout erythroid cells.

The herein described approach improves upon existing ones to edit blood groups. Some individuals have successfully been transfused with RBCs made ABO-compatible through glycosidase treatment [48–50], but this strategy is restricted to the ABO system. Blood groups have also been genetically modified in HSPCs through lentiviral delivery of shRNA or cDNA [28, 45]. Recent genome editing methods, especially CRISPR-Cas9, may be more efficient than the shRNA gene knockdown and may be considered for clinical applications, in contrast to cDNA gene addition methods rather used for blood group modeling and as controls for serology laboratories [45, 51, 52]. Moreover, blood groups have been genetically modified using CRISPR-Cas9 and TALEN in immortalized erythroid cell lines and induced pluripotent stem cells [34–36], but these cells cannot reproduce the terminal steps of erythropoiesis (although their unlimited supply is a notable advantage) [53]. To address these limitations, we used a virus- and selection-free strategy to produce Rh_null_ and group O erythroblasts starting from HSPCs. The fact that a process will be used for cRBC mass production and is nearing clinical development using this cell source (Erypharm) and that our method could be compatible with it makes it interesting [54].

Many chronically transfused patients, such as those with SCD, are a conundrum in transfusion medicine. These patients often have uncommon *RH* alleles, which makes it challenging to match for all alleles and can result in alloimmunization if sub optimally compatible blood is transfused [24]. The needs of these patients could be addressed by Rh_null_ blood—a blood type that is universally compatible as it completely lacks Rh antigen expression [31]. Additionally, as Rh_null_ individuals will readily produce alloantibodies against high-prevalence Rh antigens when exposed to Rh + blood, they can only receive blood from other Rh_null_ individuals or from autologous donations. However, Rh_null_ individuals are exceedingly rare, and their RBC phenotype makes them prone to hemolytic anemia and spherocytosis [55], so that many may not qualify for blood donations. Having a complementary source of Rh_null_ RBCs would therefore greatly improve the supply of Rh_null_ blood.

We show that levels of indels ranging from 60%–85% when targeting *RHAG* in HSPCs resulted in sufficient antigen removal to recapitulate Rh_null_ serotype by hemagglutination. To explore the applicability of the approach for other blood group systems, we also targeted an *ABO* region to produce group O erythroid cells, thus maintaining H antigen expression and avoiding a Bombay phenotype [35, 56]. The resulting erythroid cells proliferated and differentiated normally and similarly to controls, as shown by the comparable proportions of CD71+, and CD235+ cells. Only 2% of erythroblasts expressed the A antigen when starting from heterozygous A/O HSPCs, whereas 16% did so when starting from homozygous A/A cells—a ploidy-dependent effect that has been observed by others when creating *CCR5* knockout CD4+ T-cells for patients with HIV [57, 58]. In a transfusion perspective, the A/O allelic status may be a relevant and practical mean to maximize gene ablation end result, since A/O individuals account for as much as 20%–30% of the general population [59–63]. Heterozygous HSPCs may thus be the preferred cell source to edit ABO in donor HSPCs bearing with other rare blood group combinations, for the specific transfusion needs of SCD patients for instance. Matching for SCD patients can sometimes be found for all other antigens such as the Kidd or Duffy (e.g. Jk or Fy a + b- or a-b + heterozygotes) but the AB antigens [13, 14]. One way to solve this could thus be to engineer O blood from donor cells bearing all other specific genotypes (such as RH, Kell, Kidd and Duffy) [21, 23].

Although the editing efficiency allowed phenotype recapitulation, some cells still expressed the Rh or ABO antigen, raising potential concerns for transfusion applications. However, residual antigen expression might be low enough to avoid transfusion reactions or alloimmunization, especially when starting from heterozygote HSPCs. While the exact threshold for this risk is unclear, the AABB standards recommend that platelet concentrates contain no more than 1% of RBCs [64], to limit the risk of alloimmunization [65]. Having more than 1% of unedited and unmatched cRBCs might thus still be slightly suboptimal, but more studies are needed to better determine this safety threshold.

Besides, Rh_null_ RBCs may serve as useful control reagents in serology since antibody identification panels do not typically contain RBCs negative for high-prevalence antigens. Alloantibodies present in a patient’s sample will thus trigger a positive reaction with all standard panels, making antibody identification challenging [47]. Rh_null_ cRBCs could be useful to serological diagnostic laboratories, making them independent from rare blood donors for testing.

In summary, we provide a proof of concept that the genes conferring human blood groups can be efficiently edited in HSPCs through CRISPR-Cas9 and successfully cultured to erythroid cells. The herein described virus- and selection-free approach complements those that started from immortalized erythroid cell lines and other delivery methods [34, 35]. The resulting cRBCs could be useful reagents in serology, or eventually address the transfusion needs of recipients with rare blood types or chronically transfused patients. Given recent developments in the mass production of cultured RBCs, this study paves the way to the production of RBCs with desirable blood compatibility features.

## Materials and methods

### Cells

Purified mobilized peripheral blood stem cells (mPBSC) were purchased from AllCells (Alameda, CA, USA). Cord blood (CB) units were collected at the Centre Hospitalier Universitaire Saint-François d’Assise (Québec, QC, Canada), after informed consent of participating mothers was obtained. A Ficoll-Paque Plus density centrifugation (ThermoFisher, Ottawa, ON, Canada) was used to recover mononuclear cells from CBs. CB CD34+ cells (CB HSPCs) were purified using the EasySep positive selection kit according to the manufacturer’s instructions (StemCell Technologies, Vancouver, bc, Canada). Purified CD34+ cells were cryopreserved in Cryostor CS10 (StemCell Technologies) and stored in liquid nitrogen until use.

### Erythroid cell culture

The previously described erythroid differentiation medium (EDM) was used for erythroid differentiation of HSPCs [40]. Briefly, purified CD34+ cells were thawed and seeded at 1 × 10^4^ cells/mL and cultured in basal medium consisting of Iscove’s Modified Dulbecco’s Medium with 2 mM glutamine, 10 μg/ml human insulin, 330 μg/ml holo-transferrin, 2 U/ml heparin solution, and 5% human plasma (ABO compatible with the donor’s cells; either a sample was taken from a donor with their consent or AB Octaplasma (Octapharma Canada, Toronto, ON, Canada) was used). Concentrations were adjusted to 1 × 10^5^ cells/ml following transfection and diluted to 2 × 10^4^–5 × 10^4^ cells/mL on day 4 and to 1 × 10^5^ cells/ml on day 8, then progressively concentrated from day 12 on (1 × 10^6^–5 × 10^6^ cells/ml). Cells were incubated at 37°C in 5% CO_2_ in a humid atmosphere.

In phase 1 (days 0–8), cells in basal medium were supplemented with 0.001 mM hydrocortisone, 5 ng/ml human recombinant interleukin 3 (IL3), 100 ng/ml human recombinant stem cell factor (SCF), and 3 U/ml human recombinant erythropoietin (EPO). In phase 2 (days 8–12), cells received 100 ng/ml SCF and 3 U/mL EPO. In phase 3 (days 12–19), cells were supplemented with 3 U/ml EPO. Day 19 of culture corresponds to cRBCs right before enucleation.

### Flow cytometry

Erythroid commitment of cultured HSPCs was characterized with flow cytometry analyses as described in Boccacci et al [39]. The antibodies were from BD Biosciences and used at concentrations recommended by manufacturer: anti-CD71-APC (transferrin receptor and erythropoiesis marker; catalog #551374), anti-CD235a-FITC (glycophorin A and late erythropoiesis marker; catalog #559943), CD47-BV421 (integrin-associated protein and part of the RH core complex; catalog #566255). The following dyes were also used: 7AAD (viability marker; Beckman Coulter, Indianapolis, IN, USA), and SYTO 13 Green Fluorescent Nucleic Acid Stain (nucleated cells; ThermoFisher). Due to increasing and very high CD235a expression levels present at the cell surface as they differentiate further into the erythroid lineage, anti-CD235a antibodies were diluted 1/125 starting from day 15 to perform cytometry analyses, to avoid clumping [39].

To determine blood group antigen expression, cells were first fixed with formaldehyde to avoid agglutination prior to labelling, then treated with primary serological anti-A and anti-B (Bio-Rad catalog #801320 and #801345), and treated with secondary anti-IgM DyLight 488 (diluted 1/20; ThermoFisher catalog # SA5–10150). The Attune NxT Flow Cytometer was used, and analyses were performed with the FCS Express™ Flow Cytometry Analysis Software (DeNovo Software, Pasadena, CA, USA).

### Nucleofection

HSPCs were thawed and incubated overnight in complete phase-1 EDM medium at 37°C incubator. On the day of transfection, 1 × 10^5^ cells per condition were centrifuged and resuspended in 20 uL P3 primary cell buffer (Lonza, Kingston, ON, Canada). The RNP complex was made by combining 100 pmol of sgRNA and 50 pmol of Cas9 protein in a 0,5 mL tube, and then incubated at RT for 10 minutes and put on ice until added to the cells. The Cas9 electroporation enhancer (Integrated DNA Technologies [IDT], Coralville, IA, USA; 100 pmol) was first added to the cells, followed immediately by the RNP complex. Cells were then subjected to the DZ100 pulse using the Amaxa 4D nucleofector X unit (Lonza). Immediately after the pulse, 100 uL of pre-warmed fresh medium was added to the cells and incubated for 10 minutes at RT. The cells were then delicately transferred in 1 mL of pre-warmed medium in a 24 wells plate and cultured as described previously [41].

### Gene editing tools

Alt-R S.p. Cas9 and sgRNAs were purchased from IDT. Guides were designed with the online tool CRISPOR [66]. The RHAG target sequence overlaps a known Rh_null_-causing SNP (RHAG*01 N.131003 G > A) [14], and the *ABO* target sequence overlaps an SNP that causes an O phenotype (ABO*O.01.01261 del G; Table S1) [14].

To assess editing efficiency, genomic DNA was prepared from 2 × 10^4^ cells—1 × 10^5^ cells with the QuickExtract™ Solution (Lucigen, Middleton, WI, USA), following manufacturer’s instructions. The regions of interest surrounding the target sites were amplified by PCR with AccuPrime Taq DNA polymerase (ThermoFisher) with the primers shown in Table S2 purchased from IDT. Samples were purified with the Illustra GFX Purification Kit (Qiagen Canada, Montréal, QC, Canada) according to the manufacturer’s instructions. For restriction-fragment length polymorphism (RFLP) assays, PCR products were digested with the KpnI restriction enzyme following the manufacturer’s instructions. TIDE and TIDER analyses were performed following Sanger sequencing of samples to assess non-homologous end joining (NHEJ) and homology-directed repair (HDR) modifications respectively (sequencing performed at Centre Hospitalier Universitaire de Québec Research Center, Québec, QC, Canada) [67, 68].

### Hemagglutination

Hemagglutination tests were performed in glass tubes with anti-A (catalog #801320), anti-B (catalog #801345), anti-H (catalog #801165), anti-D (catalog #802032), anti-C (catalog #802282), anti-c (catalog #802348), anti-E (catalog #802331), and anti-e (catalog #802372) (Seraclone ABO and Rh grouping reagents from Bio-Rad). Briefly, 2 × 10^6^ cells were added to 50 ul of antibodies and incubated at room temperature (RT) for 15 minutes before observation. Photos were taken 15 minutes after adding antibodies to 2 × 10^6^ cells.

### Statistical analysis

Paired t-tests were used to assess CD47 expression differences between non-edited and *RHAG*-edited conditions for each CD34+ cell donor (associated with Rhnull phenotype). Analyses were carried out using Graphpad Prism version 6 (Graphpad Software, CA, USA).

## Supplementary Material

Supplemental_Figures_HMG-2024-OA-00856_Boccacci_ddaf040
