# Supplementary material for: CRISPR-Cas9-driven antigen conversion of clinically relevant blood group systems
Source: Hum Mol Genet. 2025 Apr 2;34(12):1001–8. doi: 10.1093/hmg/ddaf040 (PMC12138342; doi:10.1093/hmg/ddaf040)
Supplement: Supplemental_Figures_HMG-2024-OA-00856_Boccacci_ddaf040 [file supplemental_figures_hmg-2024-oa-00856_boccacci_ddaf040.pdf]

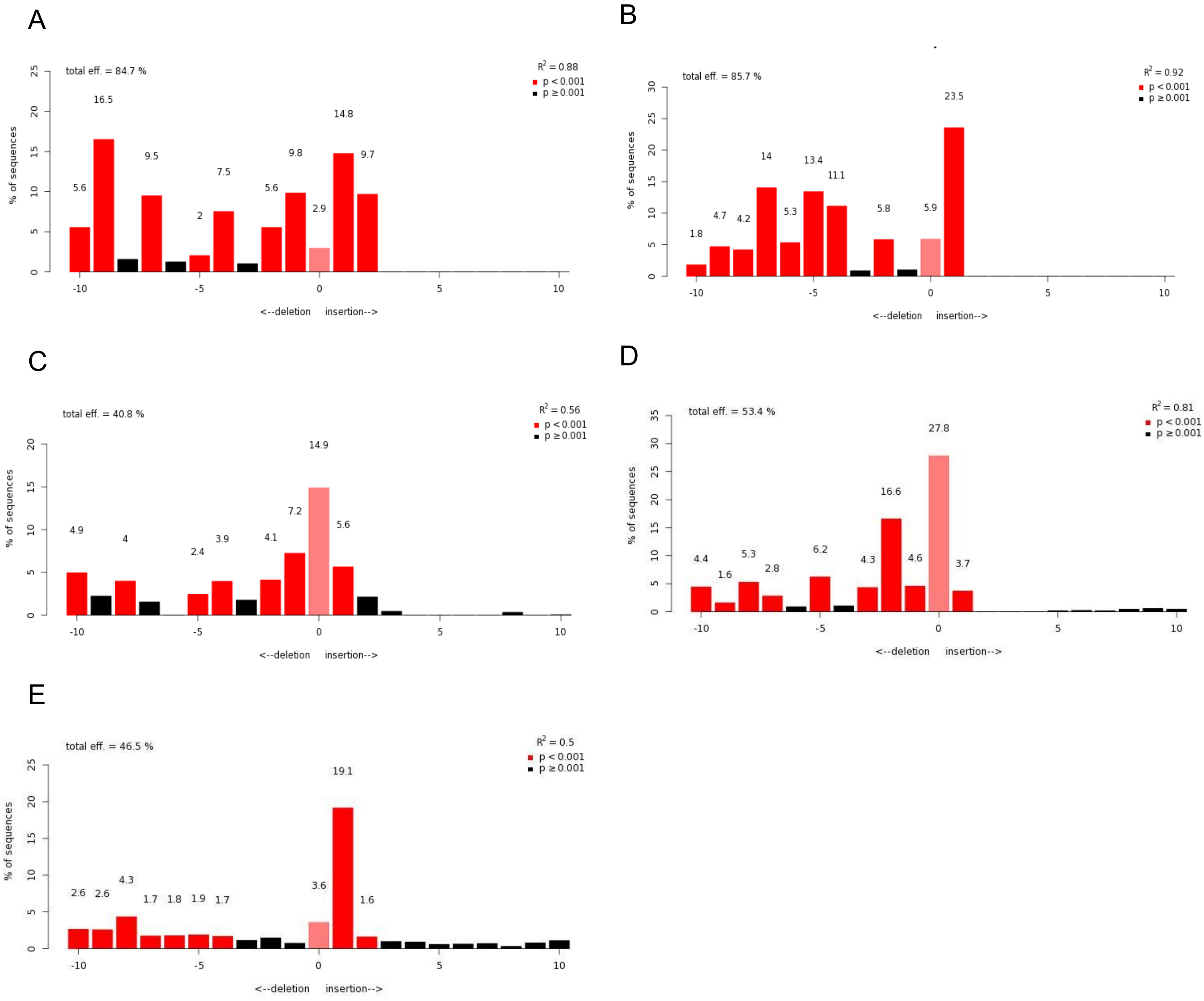

Figure S1: Screening of RHAG CRISPR-Cas9 sgRNAs efficiencies.
CB HSPCs (CB#2) were cultured in EDM and transfected with the 5 different RNPs targeting RHAG. Genomic DNA was extracted at day 4 and NHEJ efficiency was assessed by TIDE. TIDE results from experiments using sgRHAG_1 to sgRHAG_5 are shown in A to E, respectively. sgRHAG_2 was selected for subsequent experiments.

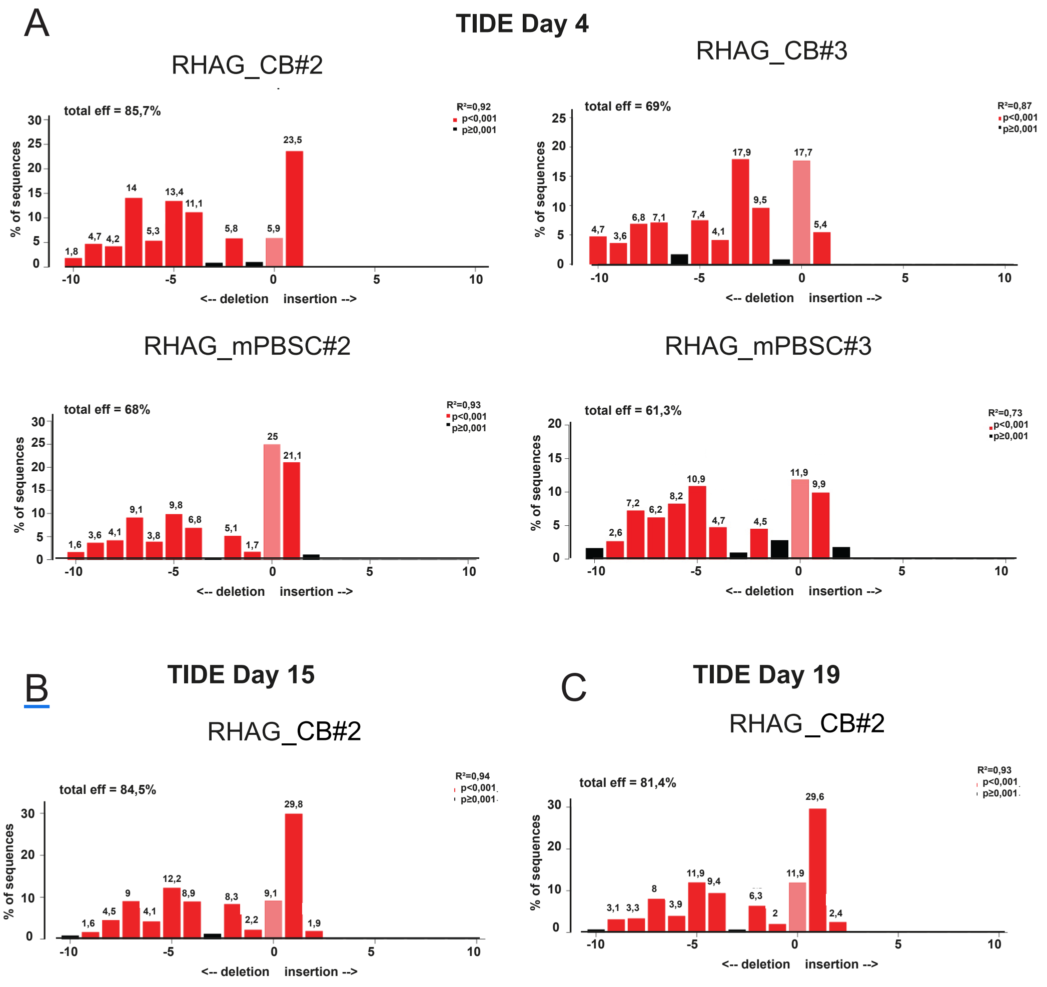

Figure S2: Efficient targeting of the RHAG gene in HSPCs.

CB and mPBSC HSPCs were cultured in EDM and transfected with RHAG_3. TIDE results of all RHAG targeting experiments are shown for day 4 (A to D), day 15 CB#2 (B) and day 19 CB#2 (C) time points.

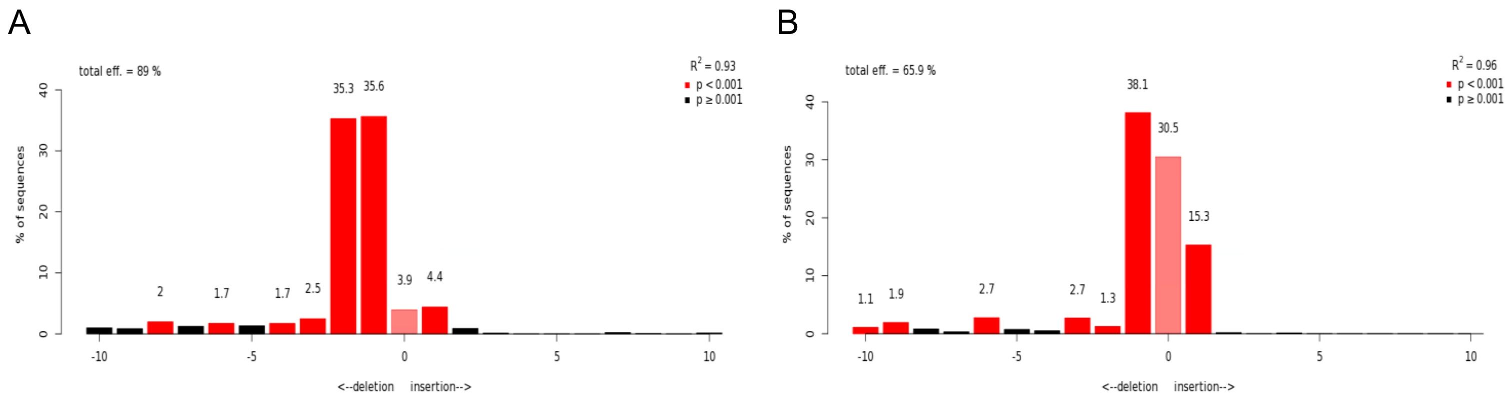

Figure S3: Screening of CRISPR-Cas9 ABO sgRNAs efficiencies.

CB HSPCs from CB#1 were cultured in EDM and transfected with different RNPs targeting ABO. Genomic DNA was extracted at day 4 and NHEJ efficiency was assessed by TIDE. TIDE results for transfections using ABO_2 (A) and ABO_3 (B) are shown. ABO_2 was selected for subsequent experiments.

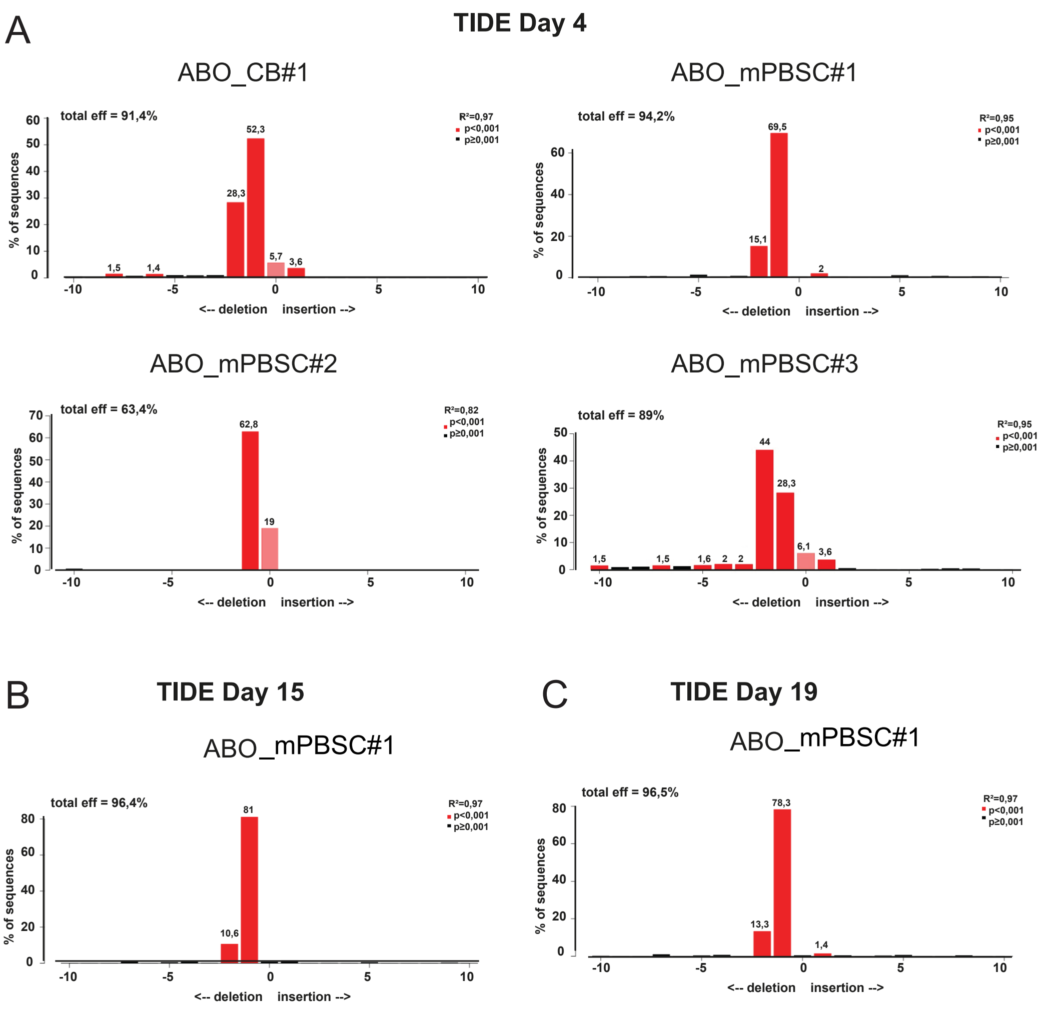

Figure S4: Efficient targeting of the ABO gene by CRISPR-Cas9 in HSPCs.

CB and mPBSC HSPCs were cultured in EDM and transfected with ABO_2. TIDE results of all ABO targeting experiments are shown for day 4 (A to D), day 15 mPBSC#1 (B) and day 19 mPBSC#1 (C) time points.

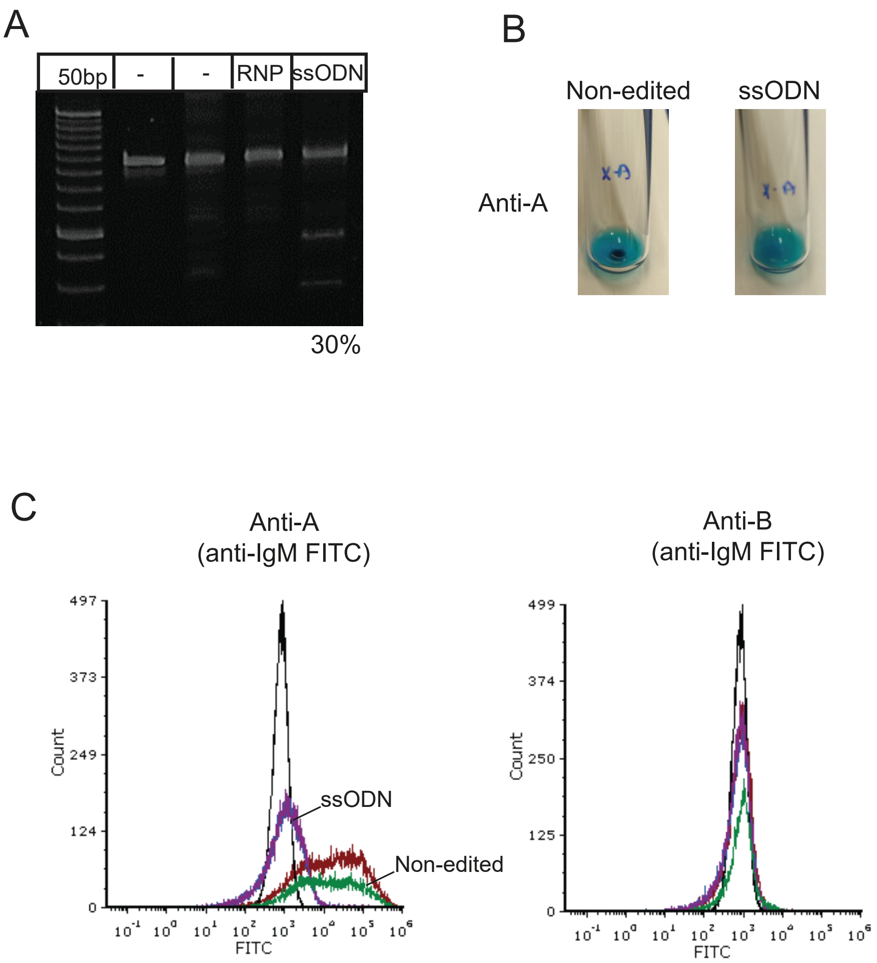

Figure S5: Assessment of an HDR-based CRISPR-Cas9 strategy in HSPCs for A to O blood group conversion of derived cRBCs.

Group A CB-derived CD34+ cells were cultured in EDM and transfected either with or without the ABO_2 RNP along with an ssODN targeting an O-associated SNP. (A) HDR efficiency assessed using RFLP at day 4; (B) Photos of hemagglutination tests performed for RNP-free and RNP-treated HSPCs at day 19 of erythroid culture;
(C) Flow cytometry analyses of A and B antigen expression at day 19.

Supplementary Tables

Table S1: Guide RNAs and ssODN sequences. *ABO* and *RHAG* sgRNA selected for experiments are shown in bold.

**Guide sequences**	
ABO_1	CCAGCCAAGGGGTCACCACG
ABO_ 2	**ATGTCCTCGTGGTGACCCCT**
ABO_3	CAGTAGGAAGGATGTCCTCG
ABO_4	CCTCGTGGTGACCCCTTGGC
RHAG_1	CCACTACACCAGGTAAGCCG
RHAG_2	**CATAACCTCCACGGCTTACC**
RHAG_3	GTACAATAGTGCCCCACTGG
RHAG_4	CCAGTGGGGCACTATTGTAC
RHAG_5	CTACACCAGGTAAGCCGTGG
**ssODN sequence**	
ABO	CACCCTGCCAGCTCCATGTGACCGCACGCCTCTC TCCATGTGCAGTAGGAAGGATGTCCTCGTGGTAC CCCTTAGCTGGCTCCCATTGTCTGGGAGGGCACA TTCAACATCGACATCCTCAACGAGCAGT

Table S2: PCR primer sequences

**PCR primer sequences**	
***ABO* Forward primer**	AGCTGGGTTTTACCGACCTG
***ABO* Reverse primer**	GGTCCAATGTTGAGGGAGGG
***RHAG* Forward primer**	ATGATCTTCTCTCAGGCGCG
***RHAG* Reverse primer**	TGGAGAACTGTGCTCTACGT
